# 

**Published:** 1886-06

**Authors:** 


hole Number, 317. JUNE, 1886. Vol. LII., Number 6.

THE

### CHICAGO MEDICAL JOURNAL AND EXAMINER

EDITORS:

S. J. JONES, M.D., LL.D.

W. W. JAGGARD, A.M., M.D. HAROLD N. MOYER, M.D.

PUBLISHED MONTHLY BY

CLARK & LONGLEY,

Under direction of

THE CHICAGO MEDICAL PRESS ASSOCIATION,

308-316 DEARBORN STREET.

